# Neural correlates of prospective memory in Parkinson’s disease: a high-density EEG study

**DOI:** 10.3389/fnagi.2026.1683562

**Published:** 2026-02-09

**Authors:** Paola Santacesaria, Stefano Vicentin, Giorgia Cona

**Affiliations:** 1 Padova Neuroscience Center, University of Padua, Padua, Italy; 2 Department of General Psychology, University of Padua, Padua, Italy

**Keywords:** ecological task, event-based prospective memory, hd-EEG, Parkinson’s disease, power spectral density, time-based prospective memory

## Abstract

**Introduction:**

Prospective memory (PM), the ability to remember and execute intended actions in the future, is a critical component of daily functioning and independent living, particularly in individuals with Parkinson’s disease (PD). Although PM deficits in PD have been widely documented, their underlying neural mechanisms remain poorly understood.

**Methods:**

This study addresses this gap by being the first to investigate the neurophysiological signatures of PM in a sample of 28 PD patients without mild cognitive impairment (MCI) and 34 matched healthy controls using high-density electroencephalography (hd-EEG). Participants completed naturalistic event-based and time-based PM tasks while monitoring virtual cooking activities embedded in a movie presented on a smart TV, with concurrent neurophysiological recording.

**Results:**

Behavioral performance did not differ between groups in either PM task, likely reflecting preserved global cognition in the PD sample; however, EEG analyses revealed marked oscillatory differences. During time-based PM tasks, PD patients exhibited increased theta, alpha, and beta power, suggesting greater engagement of internal attention monitoring and proactive control mechanisms. Conversely, during event-based PM tasks, PD patients showed reduced power in these frequency bands, consistent with a shift toward externally driven attention to monitor the occurrence of the PM event.

**Discussion:**

This pattern of findings can be interpreted within the framework of the Attention to Delayed Intention (AtoDI) model. Overall, the present study demonstrates that electrophysiological measures can detect subtle neural alterations in the absence of overt behavioral impairments and can reveal compensatory mechanisms adopted by PD patients to cope with PM demands.

## Introduction

Parkinson’s disease (PD) is a prevalent neurodegenerative disorder with an increasing global incidence (Wolff et al., 2023). The disease is driven by the accumulation of misfolded α-synuclein, which results in the progressive degeneration of both the dopaminergic striatonigral system—responsible for the core motor symptoms—and other neuronal systems and organs, causing also non-motor symptoms. While its hallmark symptoms are motor-related, cognitive impairments in PD are more variable and often subtle, particularly in the early stages, making them challenging to detect (Jellinger, 2022; Leroi et al., 2012). Given the clinical significance of non-motor symptoms, ongoing research is focused on developing more sensitive tools to enhance the early identification and characterization of cognitive deficits in PD.

Among the non-motor symptoms, prospective memory (PM) represents a particularly challenging phenomenon. PM refers to the ability to remember to carry out an intended action in the future. This capacity is of critical importance for the effective management of daily activities, the preservation of independence, and the adherence to medication schedules, ensuring the maintenance of optimal health and quality of life (Henry, 2021). PM can be classified as either event-based or time-based. Event-based PM involves the retrieval of a predefined intention when an external cue triggers it, whereas time-based PM requires the individual to self-retrieve an intention at a specific time point (McDaniel and Einstein, 2000).

A substantial body of literature has demonstrated that individuals with PD may encounter significant challenges with PM, which can further complicate their ability to effectively manage the demands of everyday tasks and responsibilities (for a review, see Costa et al., 2018). PM is a multifaceted construct involving not only executive functions but also timing and planning processes (Kliegel et al., 2005, 2011). Studying PM in PD is particularly relevant because PM deficits have been observed even when executive functions appear preserved. Moreover, PM performance correlates with autonomy in daily activities (Costa et al., 2015; Pirogovsky et al., 2012; Woods et al., 2012) and is associated with timing deficits, a distinctive feature of cognitive decline in PD (Parker et al., 2013; Raskin et al., 2011). Thus, investigating PM as a separate construct is important to fully understand its impact on functional autonomy beyond what can be explained by general executive dysfunction.

Several studies have demonstrated event-based PM impairment in PD, which may be a consequence of reduced ability to self-initiate intentions (Costa et al., 2008; Kliegel et al., 2011; Pagni et al., 2011). Costa et al. (2013) investigated the role of both retrospective and prospective components in PM performance in patients with PD. The retrospective component of PM entails the recollection of the specific content of an intention (i.e., the action that must be carried out at a future point in time). To assess the contribution of the retrospective component, the authors manipulated the memory load by varying the number of target words (one vs. four). The findings revealed that patients with PD exhibited poorer performance than healthy controls in all PM conditions, irrespective of the retrospective memory load. This indicates that the reduction in PM observed in patients with PD cannot be attributed solely to impairments in retrospective memory. The authors also proposed that a severe attentional deficit in PD patients does not fully account for the dysfunction observed in the prospective component of memory, which involves the ability to activate the intention at the appropriate moment. Specifically, increasing the attentional demands of the ongoing task did not significantly affect PM performance in PD patients across any of the tested conditions. Furthermore, their ability to complete the ongoing task was comparable to that of healthy controls. This indicates that, although retrospective memory plays a role, altered self-retrieval processes related to the prospective component of PM might explain the reduced PM performance in PD patients.

Other studies have reported that PD is associated with a disproportionate deficit on time-based as compared to event-based PM, given the fact that time-based tasks rely heavily upon the frontal system, which is commonly deteriorated in PD patients. Evidence from cognitive training studies also suggests that interventions targeting executive functions can improve PM performance, particularly in time-based tasks (Fiorenzato et al., 2025). These studies also demonstrated a significant interaction between such impairment and deficits in executive functioning and working memory (Costa et al., 2008; Raskin et al., 2011). It has been proposed that difficulties in working memory are associated with an impairment in the phase of intention formation in PD patients, which ultimately results in a deficit in PM performance (Jia et al., 2018). However, in their review of the literature on PM in PD, Costa et al. (2018) found no differences between time-based and event-based PM tasks performances. Furthermore, the authors presented evidence indicating that PM is not impaired in all individuals with PD, but only in those with mild cognitive impairment (MCI). This finding underscores the importance of assessing PM in individuals with PD to identify those with MCI. While PM deficits have been consistently linked to the presence of MCI in PD, emerging evidence indicates that subtle cognitive changes may already be present in PD patients without MCI, particularly in executive and memory functions that are critical for PM (Costa et al., 2018).

Examining the neurophysiological correlates of PM in cognitively intact PD patients may therefore help to detect early signs of cognitive vulnerability and clarify which PM alterations occur before the onset of overt cognitive impairment. A comprehensive understanding of the cognitive burden associated with PD requires an in-depth study of the impact of PD on PM and its neural mechanisms. To this regard, the neural underpinnings of cognitive impairment in PM processes in PD remains poorly understood. In particular, no study has ever explored EEG changes in PM associated with PD. In general, few studies employed EEG to investigate the neurophysiological features linked to cognitive dysfunction (Wang et al., 2020). PD-related alterations in P300 amplitude and latency were linked to declines in attention and cognitive functioning (Georgiev et al., 2015; Maidan et al., 2016). Additionally, PD patients exhibited increased alpha and beta power during both single- and dual-task conditions compared with age-matched healthy adults. Furthermore, reduced delta power was observed during dual tasks, indicating that additional modulation of readiness and attention is necessary in elderly individuals and those with PD, resulting in greater recruitment of brain resources (Possti et al., 2021). Also, in recent years, resting state EEG data were employed to investigate possible biomarkers for cognitive decline in PD patients (Arnaldi et al., 2017; Caviness et al., 2015, 2016; Cozac et al., 2016; Schlede et al., 2011; Utianski et al., 2016). Resting-state EEG studies revealed alterations in alpha and theta oscillations in individuals with PD, particularly a reduction in alpha peak frequency and an increase in alpha and theta activity in those without dementia (Sinanović et al., 2005; Soikkeli et al., 1991; Ye et al., 2022). Altered resting-state alpha and theta oscillations were found to correlate with general cognitive decline (Klassen et al., 2011; Zimmermann et al., 2015).

To the best of our knowledge, this is the first study to investigate EEG features of PM in a cohort of PD patients without MCI, with the aim of exploring neural oscillatory patterns by employing high-density electroencephalogram (hd-EEG).

It has been shown that theta (4–7 Hz), alpha (8–12 Hz) and beta oscillations (13–30 Hz) in healthy adults play a fundamental role in PM processes, particularly in the allocation of attention toward cues associated with intention and toward internal representation of intention (Cona et al., 2020; Laera et al., 2021; Vicentin et al., 2024, 2025). These studies employed classical laboratory paradigms and focused exclusively on event-based PM tasks, thereby prompting the necessity to implement more naturalistic designs that also include time-based conditions. PM difficulties observed in laboratory-based tasks do not necessarily generalize to impairment in everyday life. Therefore, ecological studies exploring the relationship between impaired prospection and functional impairment in everyday life represent a vital, but yet unexplored area within this field (Irish and Piolino, 2016).

In this vein, we obtained electrophysiological data from PD patients with no cognitive impairment and age-matched healthy individuals while they were engaged in a PM naturalistic task comprising a time-based and an event-based conditions. The study was designed as an exploratory analysis, as no hypotheses were preregistered, to map potential neural oscillatory correlates during a PM task, specifically targeting frequency bands previously associated with intention maintenance and retrieval processes in PM.

## Materials and methods

### Participants

The study sample consisted of two groups matched for age and education: 30 older adults with a diagnosis of Parkinson’s disease (PD group; mean age = 70.12 ± 6.47 years; 16 males; mean disease duration = 6.34 ± 4.03 years) and 38 healthy older adults without psychiatric or neurological diagnoses (HO group; mean age = 70.06 ± 5.20 years; 17 males). PD patients were in the “on” state for antiparkinsonian medication. All participants had normal or corrected-to-normal visual acuity and hearing and exhibited cognitive integrity. In addition, a cognitive assessment was performed prior to the beginning of the experiment using Addenbrooke’s Cognitive Examination III (ACE-III), a brief cognitive screening instrument that assesses attention/orientation, verbal fluency, memory, language, and visuospatial abilities (Pigliautile et al., 2019). The raw score was adjusted for age and education. All participants who achieved a score above the cut-off (68.68) were included, following the criteria established by Pigliautile et al. (2019). Based on the MDS PD-MCI diagnostic criteria and Level I (abbreviated assessment) guidelines (Litvan et al., 2012), which require impairment in at least two cognitive domains, none of the PD participants meet the criteria for PD-MCI. Among the total sample, two participants were excluded due to their inability to understand the instructions and correctly perform the task. Three participants of the HO group were excluded due to the excessive number of electrical or biological artifacts (gross movements, high muscular tension) in their EEG recordings. In the PD group, one participant was excluded due to the revision of its diagnosis (from idiopathic PD to Progressive Supranuclear Palsy) after the experiment was administered. The final population comprised 28 participants in the PD group and 34 in the HO. Prior to their participation, all participants expressed their informed consent. The experiment was approved by the Ethics Committee of the Department of General Psychology at the University of Padua and followed the guidelines of the Helsinki Declaration.

### Procedure

Data were collected in the EEG laboratory of the Veneto Institute of Molecular Medicine (Padua). The total duration (including cognitive assessment, EEG preparation, and the experimental paradigm) was approximately one hour and a half. The experiment was presented on a 15-inch display and participants were positioned on a comfortable chair approximately 60 cm distant from the screen.

**FIGURE 1:**
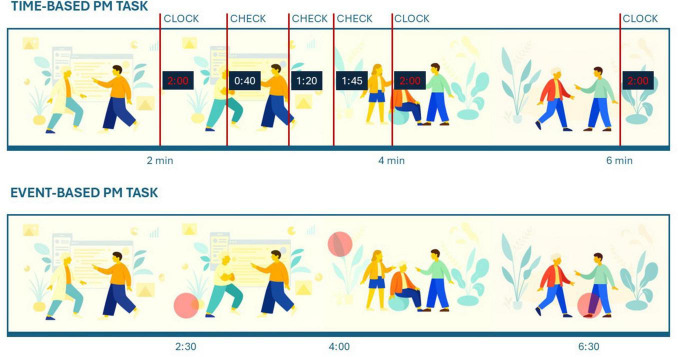
Schematic representation of the experimental design. Participants performed two prospective memory conditions while watching a continuous video. In the time-based condition (top panel), the target action was executed every 2 min. When pressing the target key, a red clock appeared at the center of the screen to confirm the elapsed interval, whereas a white clock appeared when checking the time during the interval. In the event-based condition (bottom panel), the intention was triggered by a red circle resembling an oven light, displayed slightly above or below the screen center; the position of the circle was associated with two different response keys. Images were created by the authors using open-source illustrations from Humaaans (https://www.humaaans.com, licensed under CC0).

The experimental paradigm was created using Psychopy (version 2022.2.4) (Peirce et al., 2019) and consisted of four distinct blocks. Its structure was modeled after that of our previous study (Santacesaria et al., 2025). The first block consisted of a 5-min resting-state condition, during which participants were asked to remain still and fixate on a cross at the center of the screen while electrophysiological data were recorded at rest. In the other three blocks, three distinct movie excerpts from “The Simpsons Movie” were presented to participants (10-min each, counterbalanced between participants). In the Passive viewing condition, participants were instructed to relax, enjoy the video, and pretend to be at home watching TV. This condition was always presented second, serving as the baseline condition to compare EEG activity in a passive viewing block with the activity associated with the addition of some prospective instructions in the following two conditions. The two following PM tasks mimicked everyday real-life activities and were presented in counterbalanced order. Each PM condition included a total of five cues, occurring on average every two minutes. The low number of PM cues per condition reflects the inherent nature of PM paradigms, which require infrequent cues during ongoing tasks to preserve ecological and theoretical validity. Participants were not informed about the duration of the movie. In the Event-based condition, participants were instructed that a cake was placed in the oven and that, while continuing to watch TV, they had to monitor for the appearance of a red circle right above or below the center of the screen, warning them that the temperature of the oven had to be either increased or decreased (pressing the upper or the bottom arrow, respectively) to prevent the cake from burning or being undercooked. The lower panel of Figure 1 presents the layout of the task screen, illustrating the red circle cues signaling the required temperature adjustments. The position of two subsequent PM cues (up, down) and the intervals between them (1.5, 2, and 2.5 min) were counterbalanced across participants. In the Time-based condition, the ongoing task remained the same (watching TV), whereas the PM task consisted of remembering to press a key every two minutes to check the progress of the cake baking. To minimize a potential confound, we explicitly instructed participants not to count and emphasized that they should rely on their natural sense of time. In accordance with the classical time-based PM paradigms, upon pressing the response key, a digital timer was displayed on the screen for two seconds, thereby enabling participants to evaluate their performance. Similarly, participants were also allowed to press a different button to monitor the passage of time. Doing so, a white digital timer appeared on the screen for two seconds (see Figure 1, upper panel). Participants could check the time for a maximum of three times within each 2-min interval, to limit movement artifacts in the EEG signal. The response and check keys (upper and bottom arrow) were counterbalanced across participants. To familiarize with the instructions, a 1-min practice version of the time-based condition was proposed to the participants before the beginning of the task. The low number of PM cues (five per condition) reflects the inherent structure of PM paradigms, which require rare cues during ongoing tasks to preserve ecological and theoretical validity.

### EEG data pre-processing

Neurophysiological data were acquired using a high-density (256 channels) EEG system (EGI, Electrical Geodesic Inc). Data were sampled continuously at 1,000 Hz with Cz as the online reference. Offline, the data were re-referenced to the average of all channels prior to preprocessing. EEG preprocessing was conducted using Brainstorm (Tadel et al., 2011). First, recordings were downsampled to 250 Hz and filtered in the 1–40 Hz band. Cheeks and outer rings electrodes were removed. Each condition was visually inspected to identify other bad channels which were then removed and interpolated. Biological and electrical artifacts were detected through visual inspection and corrected either removing bad segments from the recording or using the ICA approach (ICA Infomax) to remove them. Specifically, recordings were decomposed into 64 components, removing an average of 22 (±5.75) components to correct muscular or electrical artifacts. Artifact-related ICA components were identified and removed through careful manual inspection of each component’s spatial topographies, power spectra, and time courses. Finally, the continuous EEG data was segmented into 3-s epochs, visually inspected to remove the ones still displaying artifactual signal.

### EEG analysis

Epochs extracted from the recording were divided depending on their distance from the PM response, and three intervals (60 s before, 30 s before and 30 s after) were considered (Mioni et al., 2020). These three intervals were analyzed separately, computing the power spectral density (PSD) using Welch’s method (window length: 3 s, overlap ratio: 50%). Analysis focused on the theta (4–7 Hz), alpha (8–12 Hz) and beta (13–30 Hz) frequency bands. The length of the three intervals was selected to ensure a sufficient number of epochs (50 per condition) and the coverage of the shortest interval that could elapse between distinct PM cues. This approach allowed us to consider the power elicited in the period preceding the cue, following the retrieval of intention and in a distant time point from the cue when strategic monitoring is at its minimum. In the final analysis, we considered only the epochs occurring around correct responses. For the passive viewing condition, we epoched the signal in accordance with the same pattern, with a reference point set at the 2-min mark. The passive viewing condition was used as a baseline, and its signal was subtracted from both PM conditions for each participant. All group comparisons were performed on EEG power contrasted against this baseline.

### Statistical analysis

#### Behavioral analysis

Behavioral data were compared between the HO and the PD groups using an independent sample *t*-tests approach. Normality of the behavioral data was assessed using the Shapiro–Wilk test prior to conducting statistical analyses. While in the event-based PM task accuracy rates and reaction times (RTs) were considered, in the time-based condition analyses focused on accuracy rates and distance from target time (the distance between the response given and the actual target time, i.e., 2 min). Responses were considered correct if the response key was pressed within a ± 6-s window around the target time. This interval was selected as it corresponds to the 10% of the total target time’s interval, in accordance with the methods of Vanneste et al. (2016) and Laera et al. (2023). In addition, to directly compare accuracy rates across task types and groups, a mixed-design ANOVA was conducted with task (event-based, time-based) as a within-subject factor and group (HO, PD) as a between-subject factor.

Additionally, the number of time checks during the time-based task was calculated and considered for the analysis. First, the association between monitoring frequency (i.e., the total number of clock checks) and accuracy rates was investigated, using a correlational approach (Spearman correlation coefficient). Second, the two-minutes interval was divided into 30-s blocks. The clock checks were counted based on the block in which they occurred. A two-way ANOVA was conducted to determine whether there were significant differences in the distribution of the time checks between the two groups.

#### EEG power analysis

The PSD computed in the event-based and time-based PM tasks were compared between the PD and the HO groups using Brainstorm. Namely, two-tailed permutation *t*-tests for independent samples were performed, separately for frequency band (theta, alpha, and beta) and time window (60 s before, 30 s before, 30 s after). The distribution of the statistics of interest was approximated using a Monte Carlo approach with 1,000 randomizations. Results were corrected for multiple comparisons using the false discovery rate (FDR) approach.

## Results

### Behavioral data

A series of Student *t*-tests for independent samples was conducted to ascertain whether there were any statistically significant differences in the behavioral outcomes observed at the two tasks between the two groups. Descriptive statistics from the Student *t*-test of accuracy rates, RTs and distance from the target time for the time-based condition comparing the PD group and the older adults’ group are summarized in Table 1. Interestingly, no significant differences were found between the two groups in none of the PM tasks.

**TABLE 1:** Student’s *t*-test for independent analyses for the behavioral outcomes of event-based and time-based PM tasks showing comparisons between the two groups.

	*t*	df	*P*	Cohen’s d	SE Cohen’s d
**Event-based PM**					
Accuracy	0.352	64	0.726	0.087	0.248
RTs	−0.822	61	0.414	−0.209	0.255
**Time-based PM**					
Accuracy	−1.203	63	0.234	−0.300	0.252
Distance from target	−1.226	57	0.225	−0.320	0.264

The analysis revealed no statistically significant difference in the accuracy percentage between the elderly group (M = 85.4, SD = 27.34) and PD patients (M = 82.76, SD = 33.69) (*p* = 0.726) in the event-based PM condition. The two groups exhibited a high and comparable rate of accuracy in performing the task. Additionally, no significant difference was observed in RTs between the two groups (*p* = 0.414). The mean RT for the older healthy group was 2.99 s (SD = 1.16), while the PD group exhibited a mean RT of 3.27 s (SD = 1.45).

Participants showed comparable performance outcomes also in the time-based PM condition. The mixed-design ANOVA indicated a significant main effect of the task, with lower accuracy in the time-based compared to the event-based condition (*p* < 0.001, partial η^2^ = 0.195). The effect of group (*p* = 0.47, partial η^2^ = 0.008) and the task × group interaction (*p* = 0.278, partial η^2^ = 0.019) were not significant. Importantly, no statistically significant difference in the accuracy percentage were shown between the elderly (M = 55.56, SD = 35.81) and PD patients (M = 66.2, SD = 65.09) (*p* = 0.234). Differences in the distance from the target time were not found between groups (*p* = 0.225). The mean time for HO was 0.344 s (SD = 1.77), while the mean time for PD patients was 0.92 s (SD = 1.92). The low accuracy percentage suggests that all participants experienced some difficulties when attempting to monitor the time-based PM intention, which ultimately resulted in a low level of PM performance. However, when the intention was correctly retrieved (i.e., to press at the passage of two minutes), all participants responded in a very close proximity to the target.

**FIGURE 2:**
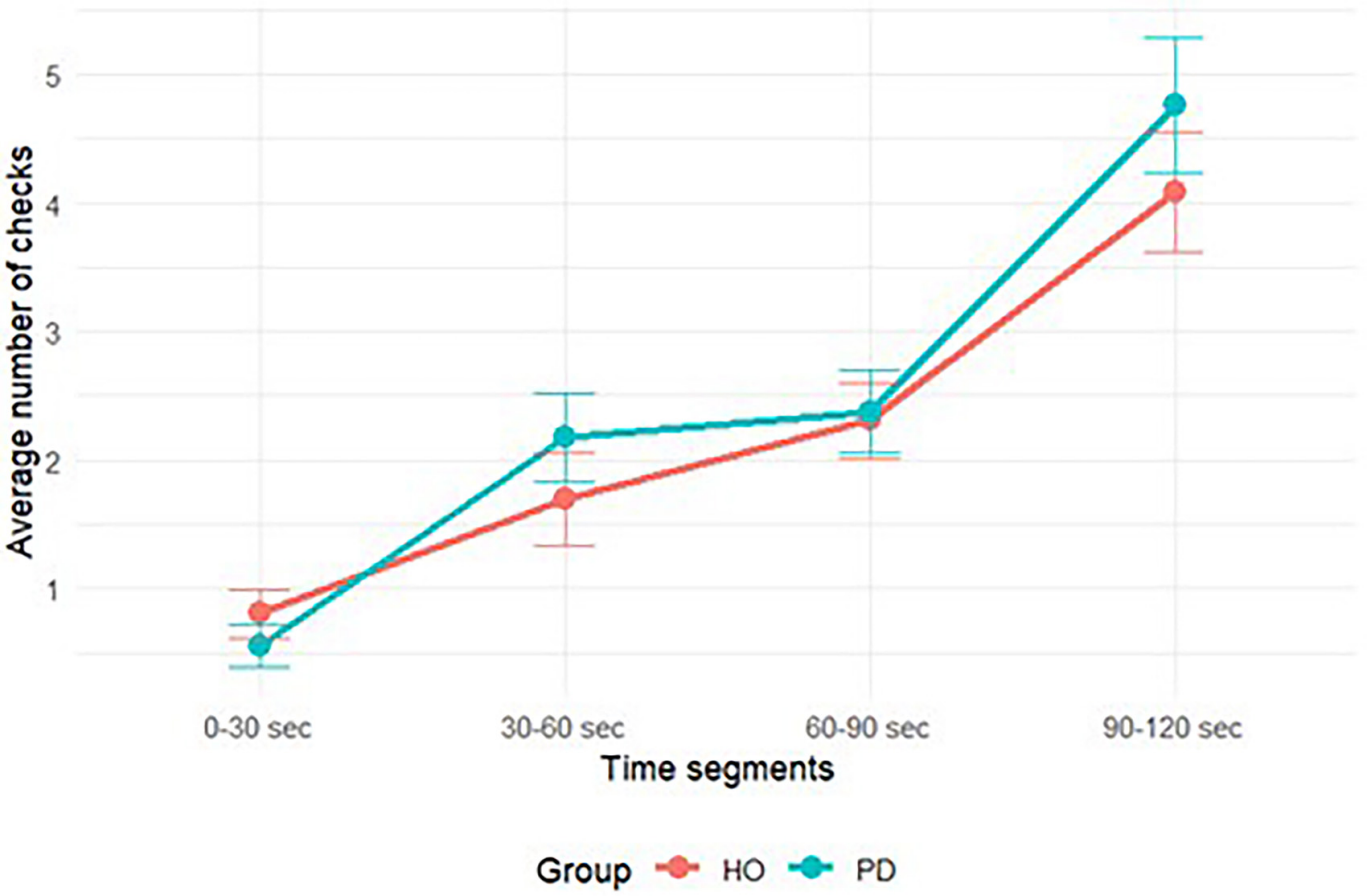
Distribution of clock-check responses across 2-min intervals for the two groups. The *X*-axis represents time segments within each 2-min interval, and the *Y*-axis shows the average number of clock checks. The blue line corresponds to the Parkinson’s disease group (PD) and the pink line to the healthy older adults group (HO). Both groups increased clock-check frequency as the interval progressed, with the PD group showing a steeper rise in the final segment (90–120 s). Error bars indicate standard errors.

The monitoring frequency in the time-based PM task was quantified by counting the number of clock checks performed within each 2-min interval (see Table 2). As illustrated in Figure 2, the distribution pattern of the clock checks exhibited a comparable linear increase over time in both groups, indicating that participants predominantly checked the clock during the final segment prior to reaching the target time, while the initial time segment saw the fewest checks. The analyses revealed no statistically significant difference between the two groups in the total number of checks, either when considering the total checks (*t* = −1.22, *p* = 0.23, Cohen’s d = −0.3), or when examining them across each time segment [*F*(1, 252) = 0.93, *p* = 0.337]. However, there was a significant main effect of time segments [*F*(3, 252) = 37.36, *p* < 0.001], indicating that the number of checks varied significantly across different time intervals. The interaction between group and time segments was not significant [*F*(3, 252) = 0.68, *p* = 0.568], suggesting that the pattern of checks across time intervals was similar for both groups.

**TABLE 2:** Means and standard deviations of the total number of checks used during the task and of the number of checks as a function of the time segment of the 2-min interval by the older group and the PD group.

	N° of checks									
	Total interval		1st block		2nd block		3rd block		4th block	
	HO	PD	HO	PD	HO	PD	HO	PD	HO	PD
Mean	8.611	9.828	0.806	0.552	1.694	2.172	2.306	2.379	4.083	4.759
SD	4.217	3.704	1.142	0.870	2.162	1.853	1.802	1.720	2.862	2.837

Moreover, both groups exhibited a statistically significant positive correlation between the total number of checks and task accuracy (HO: *p* < 0.001, *r* = 0.6; PD: *p* = 0.002, *r* = 0.54). Those who enhanced their capacity to track elapsed time showed greater accuracy in their performance (Table 3). Further analysis of the correlation between accuracy and monitoring frequency across different time blocks revealed a negative correlation between accuracy and the number of checks made within the first segment of each 2-min interval in both groups (HO: *p* < 0.001, *r* = −0.53; PD: *p* = < 0.001, *r* = −0.63). Conversely, checks made during the second block (HO: *p* = 0.691, *r* = 0.07; PD: *p* = 0.632, *r* = 0.09) showed no significant correlation with accuracy of the time-based PM task for both groups. The third block of the 2-min interval was found to positively correlate with accuracy only for HO (*p* = 0.019, *r* = 0.4). Notably, the number of checks utilized as approaching the PM cue showed a statistically significant correlation with accuracy for both elderly and PD patients (HO: *p* < 0.001, *r* = 0.8; PD: *p* ≤ 0.001, *r* = 0.77) (see Table 3). Those who increased the intensity of time monitoring toward the conclusion of the interval demonstrated enhanced accuracy in task performance. These findings indicate that a strategic monitoring of time becomes crucial to correctly retrieve a time-based intention. The imposition of a limit on the use of clock checks prompted all participants to employ these aids closer to the target time, thereby increasing the probability of achieving precision.

**TABLE 3:** Pearson’s correlations between accuracy at the time-based PM task and the number of checks used in the distinct blocks of each 2-min interval.

	Pearson’s *r*		*P*	
Time-based PM	HO	PD	HO	PD
Accuracy–N° total checks	0.559***	0.542**	< 0.001	0.002
Accuracy–N°checks 1st block	−0.534***	−0.631***	< 0.001	< 0.001
Accuracy–N°checks 2nd block	0.070	0.093	0.691	0.632
Accuracy–N°checks 3rd block	0.394*	0.125	0.019	0.517
Accuracy–N°checks 4th block	0.792***	0.769***	< 0.001	< 0.001

*p < 0.05, **p < 0.01, ***p < 0.001.

### EEG power data

**FIGURE 3:**
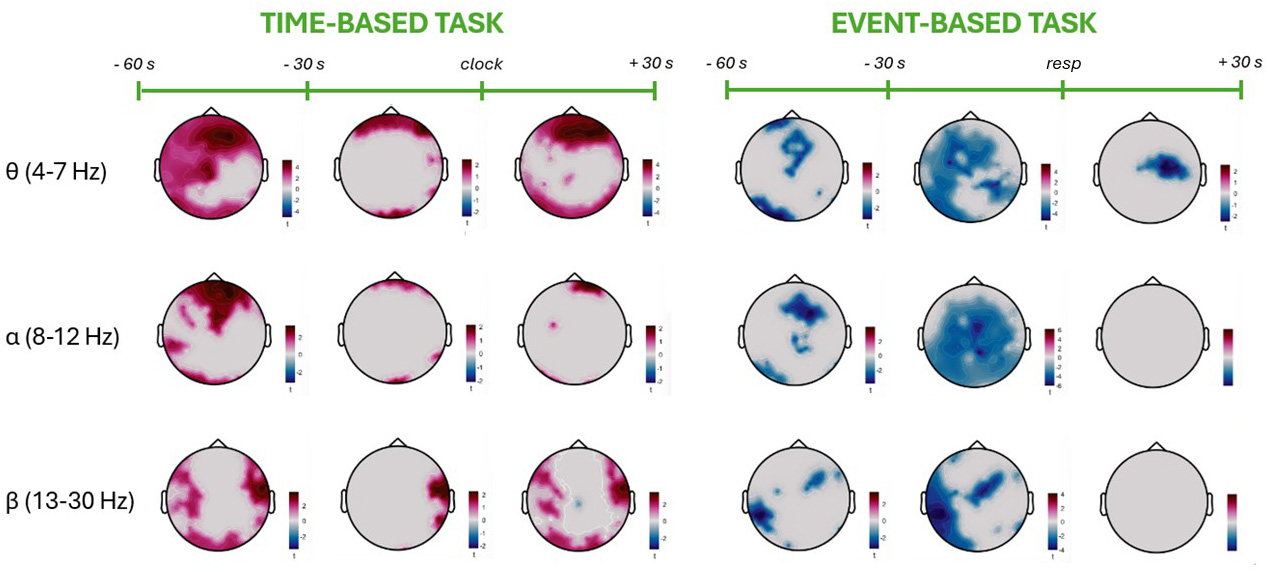
Scalp topographies of power spectral density (PSD) differences between Parkinson’s disease patients and healthy older adults during prospective memory tasks. The left panel shows the time-based task and the right panel the event-based task. For each task, three maps per frequency band (θ: 4–7 Hz; α: 8–12 Hz; β: 13–30 Hz) represent PSD differences averaged over consecutive intervals surrounding the target action, as indicated on the timeline (e.g., –60 to –30 s, –30 s to target, and target to +30 s). Red indicates higher PSD for Parkinson’s disease patients relative to healthy older adults, and blue indicates lower PSD. Group comparisons were performed on values corrected by subtracting the passive viewing condition from each PM condition for each participant, using absolute subtraction at the electrode level.

Between-group comparisons (healthy older adults vs. PD patients) were conducted separately for each 30-s time window (60–30 s before, 30–0 s before, and 0–30 s after the event-based or time-based PM response) and for each PM condition, contrasted against the baseline passive viewing condition. Power spectral density (PSD) values were grouped and averaged within canonical frequency bands: theta (4–7 Hz), alpha (8–12 Hz), and beta (13–30 Hz). As compared with the control group, PD patients exhibited an increase in all the frequency powers in time-based PM tasks (see Figure 3, left panel). Specifically, a frontal theta increase was observed across the three time-windows, with an involvement of central and left temporal electrodes in the farthest time window from the PM response. Alpha power was increased in frontal and central areas in PD patients in the farthest time window from the PM response. As the “time-target” approached, the increase in alpha power became more segregated to frontal sites. Finally, the PD group exhibited higher beta power over temporal electrodes sustained over the monitoring phases.

The comparison between the two groups in the event-based condition revealed significant clusters in all the three time-windows across all the frequencies of interest (see Figure 3, right panel). In particular, PD patients showed a theta power decrease, widespread over all the electrodes before the PM response, and more localized over central electrodes after the response. Indeed, approaching the PM response, significant differences were observed in the occipital and frontal electrodes between the two groups within this frequency band. Conversely, following the PM response, only central electrodes exhibited a decline in power in PD patients. Furthermore, a decrease in alpha power was observed in the PD group over central and posterior regions, only in the time windows preceding the PM target. In these time windows, a decrease in beta power was also found in PD patients compared with elderly group. The beta decrease was shown over posterior frontal and temporo-parietal sites. Interestingly, the decreases in alpha and beta power observed in the PD group during the time windows preceding the PM response disappeared following its completion.

## Discussion

The present study is the first to examine behavioral and neurophysiological correlates of time- and event-based PM in PD. PM deficits, common in neurodegenerative disorders such as PD, significantly affect daily functioning by compromising treatment adherence and everyday activities (Foster et al., 2009; Henry, 2021; Pirogovsky et al., 2012; Raskin et al., 2011), making it essential to clarify their neural underpinnings in ecologically valid contexts. To this end, we adopted a naturalistic paradigm involving an ongoing task (watching movie clips on a “smart TV”) and two PM conditions developed in previous work (Santacesaria et al., 2025). The time-based task required to execute specific actions at regular intervals (every 2 min), whereas the event-based task was triggered by specific visual PM cues.

No behavioral differences emerged between groups in either PM task, with comparable accuracy and reaction times. This likely reflects the preserved cognitive status of participants, confirmed by ACE-III screening prior to testing. Indeed, PD participants did not meet diagnostic criteria for PD-MCI (Litvan et al., 2012), supporting the hypothesis that PM deficits in PD typically co-occur with mild cognitive impairment (Costa et al., 2015, 2018). However, the absence of clinical variables such as motor symptom severity and PD subtypes should be considered when interpreting these findings. Prior evidence suggests that tremor-dominant PD may pose greater challenges for time-based PM compared to rigidity/bradykinesia dominance (D’Iorio et al., 2019). Stratifying patients by motor subtype could therefore yield insights not captured in the present study.

The ecological validity of the paradigm may also have contributed to high PM performance. Previous studies indicate that older adults benefit more from naturalistic contexts than laboratory settings (Koo et al., 2021; Schnitzspahn et al., 2020), possibly facilitating intention retrieval and execution in both groups.

In the time-based PM condition, both groups adopted a strategic monitoring pattern, with fewer checks initially and increased monitoring as the target approached. This distribution aligns with evidence that such strategies enhance time-based PM accuracy (Kvavilashvili and Fisher, 2007; Labelle et al., 2009; Mioni et al., 2017, 2020; Vanneste et al., 2016). Accuracy correlated with monitoring frequency during the two-minute interval, particularly between early and late phases, with no group differences in check frequency. These findings suggest that both PD patients and healthy older adults benefited from external time monitoring, which supports accurate time estimation and PM performance. Despite the absence of behavioral differences, this pattern may reflect high engagement and task compliance, potentially masking subtle impairments.

The lack of behavioral effects alongside neural disparities underscores EEG’s potential as a sensitive tool for detecting early neural alterations before measurable behavioral changes occur. Indeed, EEG power analysis revealed marked differences between the PD and control group and specific patterns for time-based and event-based PM tasks. In the time-based PM condition, PD patients exhibited increased anterior theta power compared with controls, particularly in the earliest time window. This finding aligns with evidence linking theta oscillations to strategic monitoring and internally directed attentional allocation involved in maintaining intentions active in working memory (Cona et al., 2020; Laera et al., 2021; Martin et al., 2007). Internally directed attention is critical for time-based PM, where continuous monitoring of elapsed time is required as hypothesized by the Attention to Delayed Intention (AtoDI) model (Cona et al., 2020; Santacesaria et al., 2025). The increase in theta power observed in PD patients may reflect a compensatory recruitment of internal attentional resources to sustain intentions, potentially indicating early cognitive vulnerability. Prior research associates increased theta with higher risk of cognitive decline (Cozac et al., 2016), suggesting these neural changes may precede behavioral impairments. Furthermore, the findings of the present study lend support to this hypothesis, as the results of the mixed-design ANOVA demonstrated that the time-based PM task was generally more demanding, exhibiting lower accuracy in the time-based compared to the event-based PM condition. However, this interpretation remains preliminary and warrants confirmation through longitudinal studies.

Heightened frontal alpha power was also observed in PD patients during time-based PM. Increase in alpha oscillations are linked to working memory retention (Jensen et al., 2002; Klimesch et al., 2007; Roberts et al., 2013). Based on the AtoDI Model (Cona et al., 2020), sustained alpha increase may reflect the effort to actively maintain intentions when internal monitoring is required in the absence of external cues. Notably, alpha engagement was broader during maximum monitoring phases and decreased as the target approached and participants relied more on clock checks, indicating that external cues progressively reduce the internal cognitive load.

Analogous patterns were observed in the beta band. PD patients showed increased beta power during time-based PM, particularly in temporal regions, and this modulation persisted across monitoring phases. Beta oscillations have been consistently associated with timing abilities and temporal estimation (D’Angelo et al., 2023; Kononowicz and van Rijn, 2015; Wiener et al., 2017), influencing both the perceived duration of intervals and their maintenance in memory (Wiener et al., 2018). In time-based PM, successful performance depends on the ability to internally track time in the absence of salient external cues. Accordingly, the engagement of beta activity observed here may reflect processes supporting internal time estimation and the maintenance of delayed intentions, a scenario that is compatible with the AtoDI model (Cona et al., 2015). The persistence of beta activity suggests a continuous allocation of resources to sustain these processes throughout the task. These findings are consistent with our previous study (Santacesaria et al., 2025), which showed that beta enhancement was specific to time-based PM, and likely reflects task-specific cognitive operations rather than general motor–cognitive demands. Finally, the restriction of clock checks in the present design may have further increased reliance on internal time estimation, making beta activity a plausible neural correlate of these processes.

Nevertheless, an alternative interpretation considers a role of beta in motor control and movement preparation (Cahn et al., 2013; Delval et al., 2018; Tard et al., 2016). Increased beta may index compensatory mechanisms supporting motor coordination, particularly for clock-checking behaviors and the execution of the PM response (Laera et al., 2021). Given that patients were tested under regular dopaminergic therapy, heightened beta could also reflect dopaminergic modulation of cortico-basal ganglia circuits, consistent with evidence of task-dependent beta expression under dopamine influence (George et al., 2013; Iskhakova et al., 2021).

Previous studies report increased alpha and beta power in PD during dual-task procedures (Possti et al., 2021), linking these oscillatory patterns to additional modulation of attentional and motor resources under higher cognitive load. PM paradigms resemble dual-task contexts, requiring intention monitoring while performing an ongoing task (Bisiacchi et al., 2009; Hicks et al., 2005). Thus, the observed alpha and beta increases may reflect the cognitive demands imposed by these dual-task requirements. However, this alternative interpretation does not account for the decrease in beta and alpha power observed during the event-based PM task. Therefore, the increase in beta and alpha power observed in PD patients is more likely to reflect a task-specific cognitive process engaged in time-based PM, rather than a non-specific ability or general condition related to motor–cognitive performance.

Overall, increased theta, alpha, and beta power during sustained monitoring in time-based PM aligns with prior EEG studies reporting elevated power during cognitive engagement (Possti et al., 2021; Wang et al., 2020) and resting-state studies showing altered alpha and theta in PD (Sinanović et al., 2005; Soikkeli et al., 1991; Ye et al., 2022). These increases may indicate additional recruitment of neural resources to support internal monitoring and cognitive control, consistent with evidence linking oscillatory changes to compensatory mechanisms and cognitive decline in PD (Georgiev et al., 2015; Klassen et al., 2011; Possti et al., 2021).

Analysis of the event-based PM condition revealed a distinct oscillatory profile compared to time-based monitoring, reflecting reliance on external environment rather than sustained internal monitoring. PD patients exhibited persistent reductions in theta power relative to controls across all time windows, with significant clusters over central electrodes and additional occipital and frontal involvement as the PM response approached. Reduced theta activity has been associated with diminished attention to internally oriented information (Cona et al., 2020; Kam et al., 2019; Magosso et al., 2021). In particular, Cona et al. (2020) demonstrated that theta increases under heightened internal maintenance demands and decreases when external monitoring is prioritized, supporting the interpretation of a shift toward externally driven attention. Similarly, PD patients showed alpha power decline during the minute preceding the PM response in event-based tasks, with no group differences post-response. Alpha reductions were evident across several scalp areas immediately before the event cue, when participants anticipated its appearance. Lower alpha is generally linked to heightened awareness of external stimuli (Cona et al., 2020), suggesting a transition toward externally oriented attention as the cue becomes predictable, in line with the AtoDI model.

Beta power also decreased in PD patients compared to controls during event-based PM, particularly over posterior frontal and temporo-parietal sites. This pattern aligns with evidence linking beta reductions to enhanced attention toward external cues (Deiber et al., 2013; Gola et al., 2013; Laera et al., 2021). Beyond attentional mechanisms, beta oscillations are involved in motor preparation and cognitive–motor integration (Cahn et al., 2013; Delval et al., 2018; Tard et al., 2016). Thus, attenuation likely reflects reduced need for anticipatory motor control when external cues guide action selection, diminishing reliance on internally driven motor planning compared to time-based PM.

Taken together, the results of the modulation observed in PD patients in time-based and event-based tasks can be indeed explained within the framework of the AtoDI model (Cona et al., 2015, 2020). According to this model, in PM tasks, attention can be directed either toward internal intentions, especially in time-based tasks, or toward external stimuli, especially in event-based PM tasks. The direction of attention has specific oscillation signatures, with alpha and theta increase associated with internal attention, to maintain active the intention in mind, and alpha and theta decreases more strictly related to external attention, required to detect the PM cue in the environment (Cona et al., 2015, 2020). Based on the AtoDI model, PD patients may compensate for difficulties in executing PM tasks by recruiting additional internally or externally directed cognitive resources, depending on the type of PM task. Specifically, in PD patients, time-based PM was characterized by increased theta, alpha, and beta activity, consistent with the allocation of compensatory internal attentional resources supporting sustained internal monitoring and proactive control. In contrast, event-based PM was associated with attenuated activity in these frequency bands, suggesting a compensatory shift toward externally oriented attentional resources, supporting processes such as cue detection (Cona et al., 2015, 2020).

Despite the novelty of this ecological hd-EEG paradigm, this study has also several limitations. Ecological validity remains constrained by the laboratory setting, despite efforts to simulate daily activities. The focus on a narrow EEG frequency range may have overlooked other relevant oscillations, such as delta, which could influence PM performance (Wang et al., 2020). As the first EEG study on PM in PD, it provides a foundation for future research, which should incorporate portable EEG devices and broader frequency analyses. Another limitation is the absence of clinical variables (e.g., disease stage, motor symptoms), which may have influenced results. Additionally, given the rarity of PM cues, correlations between EEG measures and behavioral performance could not be computed, limiting insights into PM functioning in PD.

In conclusion, this study advances understanding of PM in PD by examining cognitive and neural correlates in a naturalistic task context. The observed oscillatory patterns offer preliminary insights into the mechanisms supporting monitoring phases of time- and event- based PM tasks, which appear consistent with theoretical accounts distinguishing between internally and externally oriented attentional processes while also remaining compatible with alternative attentional or dual-task frameworks. Future research should further explore these dynamics and their implications for everyday functioning in this population.

## Data Availability

The data supporting the findings of this study are not publicly available but may be provided by the authors upon reasonable request.
